# Perceptions of adult trauma patients on the acceptability of text messaging as an aid to reduce harmful drinking behaviours

**DOI:** 10.1186/1756-0500-7-4

**Published:** 2014-01-04

**Authors:** Bridget Kool, Emily Smith, Kimiora Raerino, Shanthi Ameratunga

**Affiliations:** 1 Section of Epidemiology and Biostatistics, School of Population Health, the University of Auckland, Private Bag 92019, Auckland 1142, New Zealand; 2 Faculty of Medical and Health Science, the University of Auckland, Private Bag 92019, Auckland 1142, New Zealand; 3 Te Kupenga Hauora Maori, School of Population Health, the University of Auckland, Private Bag 92019, Auckland 1142, New Zealand; 4 Section of Epidemiology and Biostatistics, School of Population Health, the University of Auckland, Private Bag 92019, Auckland 1142, New Zealand

**Keywords:** Cellular phones, m-health intervention, Alcohol drinking, Brief intervention for harm (BI), Accidents

## Abstract

**Background:**

Brief interventions (BIs) have been shown to be effective in modifying hazardous drinking behaviours in a range of settings. However, they are underutilised in hospitals due to resource constraints. We explored the perspectives of admitted trauma patients about the appeal, acceptability and content of a Brief Intervention (BI) delivered via text messages.

**Methods:**

Thirty mobile phone users (≥16 years old) admitted to Auckland City Hospital as a result of injury were recruited (December 2010 – January 2011). Participants were interviewed face-to-face during their hospital stay using a semi-structured interview guide that explored topics including perceptions of the proposed intervention to reduce hazardous drinking and related harm, and perceived acceptability of an m-health program. Where issues relating to content of messages were raised by participants these were also captured. In addition, a brief survey captured information on demographic information, mobile phone usage and type of phone, along with the frequency of alcohol use.

**Results:**

22 of the 30 participants were male, and almost half were aged 20 to 39 years. The majority of participants identified as New Zealand Europeans, six as Māori (New Zealand's indigenous population) and of the remainder two each identified as Pacific and of Asian ethnicity. Most (28/30) participants used a mobile phone daily. 18 participants were deemed to be drinking in a non-hazardous manner, seven were hazardous drinkers, and three were non-drinkers. Most participants (21/30) indicated that text messages could be effective in reducing hazardous drinking and related harms, with more than half (17/30) signalling they would sign-up. Factors identified that would increase receptiveness included: awareness that the intervention was evidence-based; participants readiness-to-change; informative messages that include the consequences of drinking and practical advice; non-judgemental messages; and ease-of-use. Areas of potential concern included: confidentiality and frequency of messages. The cultural relevance of the messages for Māori was highlighted as important.

**Conclusions:**

This study indicates that trauma patients recognize potential benefits of mobile-health interventions designed to reduce hazardous drinking. The feedback provided will inform the development of an intervention to be evaluated in a randomised controlled trial.

## Background

The harms associated with alcohol consumption are a leading public health concern worldwide. Approximately 2.5 million deaths annually and around 5% of the global burden of disease and injury are attributed to alcohol [1]. Problem drinking is recognized as the leading risk factor for death among men aged 15 to 59 years [1]. In New Zealand, 4% of deaths annually are attributed to alcohol, with injury being the largest contributor [2]. Up to a third or more of injury-related emergency department (ED) admissions are also associated with the acute use of alcohol [3-5].

BI is a commonly used technique used to help reduce alcohol misuse, typically among non-dependent drinkers in healthcare settings [6]. The technique uses a systematic and focused approach that relies on rapid assessment of a person’s drinking risk level, quick engagement of the person, and immediate implementation of change strategies [7]. BIs provide an opportunity to screen and intervene during an obvious ‘teachable moment’ that accompanies an admission to hospital when patients are likely to be more willing and receptive to discuss their alcohol use and abuse [8,9].

Numerous experimental studies have confirmed the effectiveness and efficacy of BI in ED settings [10]. Nilsen et al. in a systematic review of BIs for injury patients in emergency care settings, reported that 11 of the 12 studies comparing pre- and post- BI results observed a significant effect on at least some of the outcomes (alcohol consumption, drinking practices, and negative consequences associated with alcohol – including injuries) [11]. A subsequent quasi-experimental study conducted in 14 sites in the US, found those receiving BI reported consuming around three less drinks per week than controls [12]. BIs have also been shown to be effective among young people attending EDs [13,14].

BIs offer the potential to reduce alcohol intake among injury cases presenting to hospital and to result in lower levels of ED/hospital re-attendance [15,16]. Despite strong evidence of their effectiveness, BIs are rarely implemented in New Zealand [17]. This is not dissimilar to the situation in the US [10,18] despite many agencies, including the US Trauma Service Guidelines [19], promoting routine screening and implementation of interventions, and most trauma patients indicating they want healthcare teams to address their problems with alcohol [8]. Foremost among identified barriers are a lack of time, resources, training, and workforce capacity [8,10,17,18,20].

The uptake of mobile phones globally is extensive with 100% saturation in developed countries and rapid increases in mobile subscriptions in the developing world from 53% at the end of 2005 to 73% at the end of 2010 [21]. Mobile network access is now available to 90% of the world’s population [21]. The delivery of health information via mobile phones (m-health) is increasingly viewed as an effective, low-cost alternative to traditional forms of information dissemination [22-27]. A recent World Health Organisation report identified mobile (and wireless) technologies as having the potential to transform the way health services are delivered globally [25]. In New Zealand, this approach has been translated into routinely delivered services for smoking cessation (for example, Txt2Quit; http://www.quit.org.nz/txt2quit/).

While the application of m-health technology to problem drinking has lagged attention compared to several other areas of behaviour modification, the transferability of the principles delivered in the form of tailored text-based interventions to reduce hazardous drinking has been demonstrated in some healthcare settings.

A US ED-based study randomised young adults identified as hazardous drinkers to receive weekly text messages with goal setting (intervention), weekly text-based drinking assessments with no feedback (assessment), or control [13]. At three months, those in the intervention group had 3.4 fewer heavy drinking days (≥ 5 drinks for men or ≥ 4 drinks for women on a single drinking day) in the last month compared to baseline, whereas in the control group the participants had only 1.1 fewer heavy drinking days (*p* = 0.04). There was good correlation between the text based queries of participant drinking consumption and the timeline follow-back method (a 28 day calendar based assessment of drinking consumption). Participants reported feeling very comfortable about sharing their drinking habits via text message, and those in the intervention group stated they found the text-messages useful in reducing their heavy drinking and would recommend the program to others who drink too much alcohol.

To the best our knowledge no studies have explored the effectiveness of m-health technology to address drinking behaviours in admitted adult trauma patients. The aim of this study was to investigate the appropriateness, appeal, and acceptability of delivering BI via text messaging to reduce harmful drinking behaviours among people aged 16 years and older admitted to hospital as a result of an injury. Where issues relating to content of messages were raised by participants these were also captured and analysed.

Whittaker et al. have identified six steps in the research and evaluation process for developing m-health interventions [27]. The current study aligns with Whittaker’s first (Formative Research) and second steps (Pretesting). In the Formative Research step, the purpose is to ‘inform the development of the intervention content and regime’. Domains covered should include: how the target group use their mobile phones, factors that would attract users to the program, and how well the intervention’s theory and evidence base fit with mobile phone delivery. In the Pretesting step, the aim is to determine the intervention’s acceptability to the target audience and to draw on the feedback to improve and refine the intervention.

This study forms the critical first phases of informing the development of an m-health intervention designed to commence following a patients discharge from hospital, the effectiveness of which will be evaluated in a randomised controlled trial. The format, frequency, and timing of the intervention will be informed by the findings of this study alongside the knowledge obtained from the published literature.

## Methods

We conducted a small mixed methods study involving semi-structured interviews and a survey of a purposively sampled group of admitted trauma patients. A purposeful sampling approach aims to select ‘information rich cases’ that yield ‘insights and in-depth understanding rather than empirical generalisations’ [28]. Therefore the sample is not intended to be representative of all patients admitted to hospital with an injury. The target population was New Zealand adults 16 years of age or older admitted to Auckland City Hospital under the care of the Trauma Service as a result of an injury during a one month period (17 December 2010 to 21 January 2011). The inclusion criteria required participants to be mobile phone users who were able to complete an interview in English or Te Reo Māori. Exclusion criteria were: cognitive deficits, pregnancy, and a history of mental illness. The ethnic groups of interest were initially defined as people of Māori (New Zealand’s indigenous population), Pacific Island and New Zealand European ethnicities. However, due to the lower than expected number of Māori and Pacific Island patients during the recruitment period, the eligibility criteria were expanded to include people of Asian ethnicity. Participation was voluntary and anonymous.

**Figure 1:**
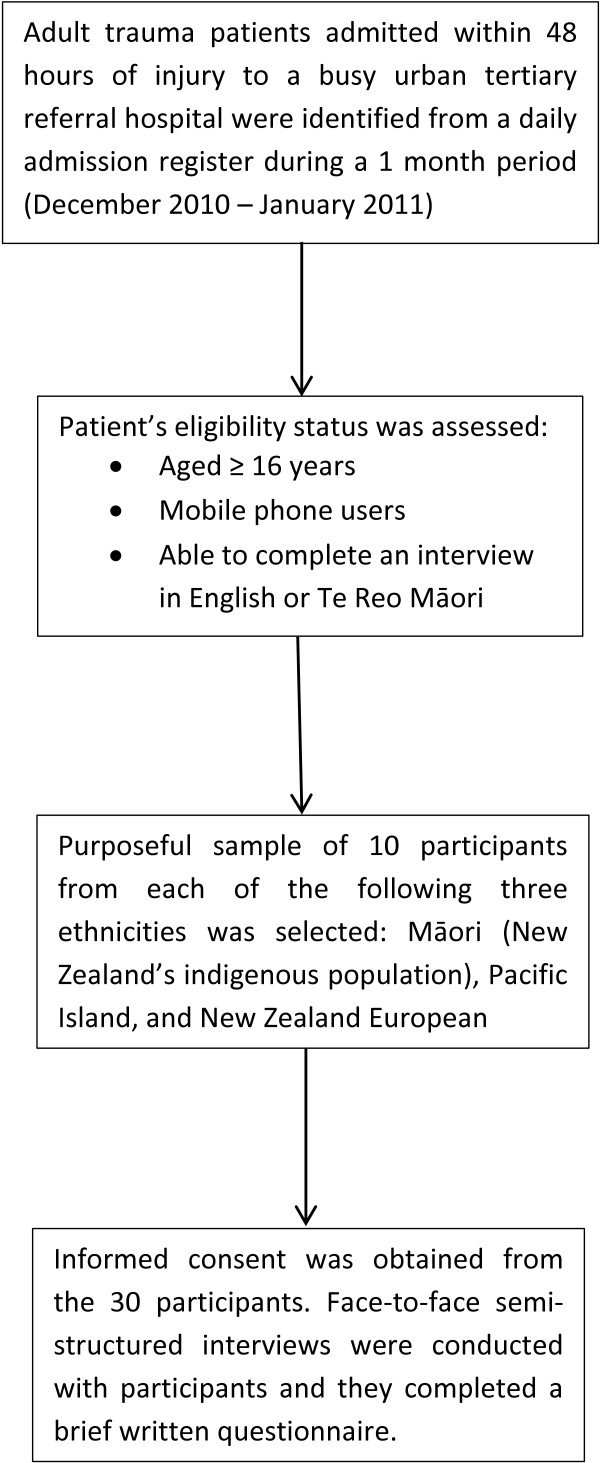
Flowchart summary of study methodology.

We aimed to recruit 30 participants, approximately 10 of each ethnic group (Figure 1). Interviews with Māori participants were conducted by a Māori research assistant, and interviews with Pacific Island participants were conducted by a research assistant of Cook Islands and European descent. Interviews with Asian participants were conducted by whichever of the two research assistants was available. During the study recruitment period, admission registers were reviewed daily Monday to Friday by the research assistants to identify potential cases meeting the inclusion criteria. Before approaching any potential participants, the research assistants checked with the trauma coordinator or patients’ nurse to ensure it was an appropriate time to visit the patient. Interviews were conducted face-to-face during the patient’s hospital admission, and were on average of 30 minutes duration.

Interviewers were blinded to the drinking status of participants at the time of the injury that resulted in their admission to hospital, unless this information was volunteered by participants during the course of their interview.

Individuals who expressed interest in taking part in the study were provided with a written participant information sheet outlining the study purpose and the overall goal of the research, participation requirements, data storage information, and contact details of the study staff including the principal investigator. They were then invited to give their informed consent to participate in the study.

Interviews were semi-structured to allow for a reflexive discussion to take place and unscripted themes to surface. Topics explored during the interviews included perceptions of the proposed intervention to reduce hazardous drinking and related harm, and perceived acceptability of an m-health program [see Additional file 1]. Specific areas explored included the perceived utility of the proposed intervention, potential negative effects, preferred source of text messages (e.g. other patients, doctors, celebrities, peers), the types of messages considered likely to be useful and possible methods of interactivity. Questions asked in the interviews were developed and reviewed by members of the research group in consultation with experts in the field. Interviews were audio-taped and transcribed with the permission of participants.

Following the interview, a survey consisting of 19 questions captured basic demographic information, mobile phone usage and type of phone, along with the frequency of alcohol use was administered. Alcohol use was classified according to the Alcohol Advisory Council of New Zealand guidelines; ‘hazardous drinkers’ are defined as males who drink more than six or females who drink more than four drinks per drinking session [29].

We did not exclude patients based on their drinking consumption patterns as we felt it was important to represent the spectrum of alcohol consumers to whom the study will be introduced in a trial setting. Therefore the acceptability of the study concept needed to be evaluated in admitted injury patients with the full range of alcohol consumption patterns.

All participants received a NZ$20 shopping voucher on completion of the interview as a token of appreciation for their participation.

Interviews were recorded and transcribed by a commercial service. The written questionnaire data was entered into a database created in Microsoft Office Excel 2007 and summarised. A General Inductive approach was used to analyse the data [30]. This approach enables a researcher to use ‘detailed readings’ of raw data to derive themes, concepts or models. The analysis of data is guided by the domains and topics to be investigated identified in the research objectives. Categories or themes are developed from the raw data and then further refined into a model or framework that contains key themes or processes. The findings emerge from multiple interpretations of the raw data by the researcher/s. The researcher makes decisions about what findings are important and less important.

In this study, responses were analysed by arranging them into upper level categories derived from the study aims: appeal of the proposed intervention, barriers and enablers to participation, factors relating to the manner in which the intervention is delivered, and where they emerged issues relating to message content. The lower-level categories were derived from multiple readings of the raw data. A process of continuing revision and refinement of the lower-level categories followed. The patterns that emerged were then synthesized. Appropriate quotations were selected that conveyed the core theme of a category. When interpreting the narratives, all project members were encouraged to debate and discuss their interpretations with each other. If consensus on the interpretation of the narrative could not be reached between the team members, the final decision would pass to the lead researcher for adjudication.

Ethical approval for the study was obtained from the Multi-region ethics committee and the Auckland District Health Board.

## Results

The results are presented in two sections; the first describes the participant characteristics and the responses to the brief survey, the second section presents the qualitative analyses.

### Participant characteristics and survey responses

Fifty-one people met the eligibility criteria for the study during the one month recruitment period. Of these, six were ineligible, six were missed (not on the ward, asleep, or busy), of the remainder 30 agreed to take part (77%). The majority (8/9) of those who refused to take part identified as New Zealand Europeans. The reasons for non-participation included: recovering from surgery (3), too tired (3), about to be discharged (1), pain (1), and not interested (1). Table 1 displays the characteristics of the 30 participants. Nearly three-quarters were male, and almost half were aged 20 to 39 years. The majority of participants identified as New Zealand Europeans, six as Māori and of the remainder two each identified as Pacific and of Asian ethnicity.

**Table 1:** **Participant characteristics in the m**-**health feasibility study** (**n** = **30**)

**Characteristic**	**Number of participants**
Age group (in years)	
20 – 29	9
30 – 39	4
40 – 49	8
50 – 59	5
60 and over	4
Gender	
Male	22
Ethnicity	
Māori	6
Pacific Island	2
New Zealand European	20
Asian	2
Employment status	
Employed	20
Student	3
Unemployed	3
Other	4
Mobile phone use	
Seldom	1
Daily	28
Unreported	1
Use of text message function	
Sometimes	2
Often	10
Always	17
Unreported	1
Mobile phone intervention targeting binge drinking appeals	
Yes	23
No	6
Unknown	1
Message format preference	
Text Message (SMS)^a^ only	15
Picture Message only	3
Video Message only	1
Text (SMS) and Picture	5
Text (SMS) and Video	1
Video and Picture	2
All	2
Alcohol drinking behaviours	
Non-drinkers	3
≤ 6 drinks /session for males (≤ females)	18
>6 drinks/session for males (>4 females)	7
Unreported	2

^a^SMS: short message service.

Most (28/30) participants used a mobile phone daily. Few participants (5/29) indicated they did not always leave their mobile phone switched on. Most participants reported using the text message function of their mobile phone 'often' or 'always' (27/29). Over half (17/29) were on pre-pay schemes for their phones. Over two-thirds (19/28) of the participants reported their phones were 3G capable.

The majority of participants (including all those of Māori, Pacific Island, and Asian ethnicity) affirmed that they would be happy to receive messages from a free health mobile phone messaging service (Table 1).

Self-reported alcohol use varied widely. Eighteen participants were deemed to be drinking in a non-hazardous manner, seven were hazardous drinkers, and three were non-drinkers. Two of the hazardous drinkers identified as being of Māori ethnicity.

### Attitudes towards using a mobile phone for addressing hazardous drinking behaviours in trauma patients

Participants reported that a mobile-phone messaging service providing information about alcohol could be effective in reducing hazardous drinking and related harms. Over half said they would sign themselves up to receive cell phone messages about alcohol. Over half of those who screened positive for hazardous drinking (4/7) indicated they would sign up for such a service.

The General Inductive analysis revealed a number of lower level categories (Table 2), these will now be described in more detail.

**Table 2:** Overview of emergent upper and lower level categories

**Upper level categories**	**Lower level categories**
Appeal of the proposed intervention	Important issue
	Evidence based
	Readiness for change
	Targeted
Barriers and enablers to participation	Ease of use
	Frequency of messages
	Respondent burden
Delivery of the intervention	Deliverer
	Text messaging features
	Timing
Intervention content	Tone of the message
	Message ideas
	Cultural relevance
	Privacy and confidentiality

#### Theme one: appeal of the proposed intervention

Generally, the intervention being proposed was well-received by participants with almost all reporting that addressing alcohol misuse was an important focus. The lower level themes that emerged in relation to the overall appeal of an intervention addressing alcohol misuse included: the significance of the issue, an evidence-base supporting the intervention, a person’s willingness to change, and the potential for targeting those who need it most.

### Significance of the issue

Some participants focused on the particular value of the intent of the intervention while others affirmed the importance of the proposed research from a knowledge generation perspective. These impressions are illustrated by the following comments, the first made by a young Māori male participant, and the second by an older European male:

“*Yeah that would be* … *pretty good*…*you might be just about to jump in your car go for a drive while you*’*re drunk and you might get one of those texts just before you do and it might make you think and stop what you*’*re doing*..” (*MM03*, *Hazardous Drinker*)

“*I would love to be a part of the randomised controlled trial because I*’*m fascinated*… *I think it*’*s a really important issue and I am fascinated about how you do it so I*’*d be very happy to participate*…” (*NE16*, *Non*-*Hazardous Drinker*)

### Evidence-based

Participants felt it was critical that the intervention was research or evidence-based, illustrated by the following statement:

“*Well if you were trying to convince me to take part in anything I*’*d want to know that it had some good sound research behind it*…” (*NE12*, *Non*-*hazardous Drinker*)

Another participant made reference to the recent m-health quit smoking trials conducted by the University of Auckland [31], commenting:

“*I don*’*t know the details but I remember there*’*s been a study on using mobile phones on smoking and I know that*’*s been very successful*… *I*’*d love to think that you know the lessons learnt from that would apply to this*”. (*MM04*, *Unknown Drinking Status*)

### Readiness to change

When participants were asked ‘What do researchers need to bear in mind to make someone like you more likely to take part in a study like the one we are proposing?’ responses commonly related to the concept of a ‘readiness for change’. An older European made the comment.

“*I think it often takes a personal thing to stimulate a strong reaction*”. (*NE04*, *Non*-*hazardous Drinker*)

These sentiments were echoed by other participants

“… *they*’*ve got to actually be in a position*, *in a state where they*’*re ready to accept their sort of action* …” (*NE17*, *Non*-*Hazardous Drinker*)

“*I think it*’*s all down to those who need it whether they*’*re going to accept it cause they*’*ve got to accept the fact that they*’*ve got a problem before they*’*ll even go anywhere near getting it fixed*”. (*NE09*, *Abstainer*)

### Targeted

Participants acknowledged a key benefit of the proposed intervention was its ability to target alcohol-related messages to those who need it most. In response to being asked ‘What other types of things would you say researchers need to bear in mind to make someone like yourself more likely to want to take part in the study that we’re proposing?’ this middle aged European man responded:

“…*I suppose target the right people not the wrong people*, *you can*’*t send out random messages you can*’*t send out to people who aren*’*t even drinking*…*so the message would go to the right person that needs to hear it*, *you could say*, *jog their memory basically that injury that was caused by in the past or something like that*”. (*NE14*, *Non*-*hazardous Drinker*)

In a similar vein, an older European male commented

“..*if we can find ways to help social groups think about those sorts of issues and respond to them that would be very helpful*” (*NE16*, *Non*-*Hazardous Drinker*)

Participants suggested that the target age bracket for this intervention should be people under thirty, although some respondents felt it would not be effective for teenagers because it would be ignored or made fun of. A point illustrated by this comment from a current university student in his early 20’s:

“*A lot of its to do with age as well like I*’*m in* (*a New Zealand tertiary institution*) *so there*’*s a bit of a drinking culture round there and as you grow older I think first of all you*’*ll be more open to this sort of stuff*, *you*’*d be more open to texts like this you know cause as a 17 or 18 year old you just say no like I don*’*t really care what they think. But now it*’*s more of a reminder saying you need to like front up to your more adult responsibilities*”. (*NE03*, *Hazardous Drinker*)

The notion of younger people caring about their friends was captured in this quote from a young Māori male:

“*I can definitely see them being helpful and I*’*ll definitely say drink responsibly and look after your mates*”. (*MM01*, *Non*-*hazardous Drinker*)

#### Theme two: barriers and enablers to participation

Lower level categories that emerged in relation to increasing or decreasing participants receptiveness to an m-health intervention targeting hazardous drinking included: incentives, ease of use, frequency of messages, and respondent burden.

### Incentives

The use of incentives was a common theme identified by participants as a way to increase the appeal of being involved in any future research regarding an m-health intervention. A young male commented:

“*Maybe rewards to get people actually involved in such a thing because a lot of people you approach and they*’*ll just say*, “*Nah*, *I can*’*t be bothered*,” *you know. But if you give them some sort of reward they*’*ll definitely come on board*..” (*NE06*, *Non*-*hazardous Drinker*)

The appeal of incentives was also noted by an older male:

“*But maybe like if you offered them a free text if they actually like read it and they send in a magic number down the bottom*, *maybe they go into the draw for something you know*, *so it*’*s an incentive for people to actually read the texts*”. (*NE18*, *Non*-*hazardous Drinker*)

“.. *maybe work out some sort of game or some sort of thing where maybe people can play each other off with their messages or whatever or they pass it on and the more you pass it on to somebody*, *they generate points type thing. So it does become a reward type programme*, *incentive scheme. But it may be after the 20th one*, *they get a free can of V* [*popular non*-*alcoholic beverage*] *or something*, *yeah*”. (*NE18*, *Non*-*hazardous Drinker*)

A counter view point was posed by this young man:

“......*for somebody that needs help and knows that they need help you probably won*’*t even need an incentive*, *the incentive would be to stop what your addiction is*..” (*MM04*, *Unknown Drinking Status*)

### Ease of use

Ease of use was signalled as an important feature of any intervention, a point made in these quotes from two middle-aged European males:

“*I*’*m not really big techno like a cell phone this is the thing*, *I mean I*’*m not one of those that goes out and buys an IPhone or whatever I mean I*’*m not that sort of person*, *you know I*’*m not in to all of that technology straight away*....” (*NE17*, *Non*-*Hazardous Drinker*)

“*It*’*s got to be easy*.....*What would make me not take part*? *Yeah*, *just if it was too hard*”. (*NE18*, *Non*-*hazardous Drinker*)

### Frequency of messages

An aspect noted and emphasized by many participants related to the frequency of messages. Participants indicated that if the messages came too frequently, these were likely to be irritating and they would be less likely to want to take part. This latter point was illustrated in the following comment which highlights some important considerations for the delivery of messages:

“*If you*’*re sending out the messages willy nilly it*’*s going to be an utter irritation to people. I think you know there*’*s some real work would need to be done in terms of how you would actually target when and how the texts would be sent because it*’*s a bit like telephone sales people calling up at dinner time on a Friday night and they I think quite often do more harm to their cause than good so that if you were constantly getting texts about not drinking you*’*re actually sitting at home not drinking*, *that would just be an utter irritation*”. (*NE12*, *Non*-*hazardous Drinker*)

The importance of balancing the need for sufficient contact to achieve the intervention goals without irritating participants was conveyed in this comment from a European male:

“..*there*’*s a point the saturation is too much* …*finding that right line that*’*s you know almost getting*, *that will get the message through without annoying them or pissing them off or something*”. (*NE14*, *Non*-*hazardous Drinker*)

### Respondent burden

Respondent burden was raised by a number of participants. A middle-aged man commented:

“*I think I probably tend to decide in my mind how much time or effort I*’*m going to give to something such as a study like this and if you were to interview me for two hours or whatever that would start to limit my enthusiasm*”. (*NE01*, *Non*-*hazardous Drinker*)

And another stated:

“*I guess time really*; *I think I would be reluctant to get involved in a trial if it was going to take up valuable free time*…” (*NE02*, *Non*-*hazardous Drinker*)

#### Theme three: delivery of the intervention

Three lower level categories emerged in relation to the delivery of the intervention. These centred on whether or not there should be a ‘face of the intervention’ and if so who, what if any text messaging features should be incorporated, and the timing of messages.

### Face of the intervention

There were mixed responses to the idea of using an ambassador or ‘face’ for the campaigns. Some participants viewed the use of a sportsperson as adding to the appeal of messages.

“..*if you had someone* … *you know have to be genuinely saying I*’*ve struggled with this and if you need a hand you can search further help on this*, *like that whole depression sort of ad you know scenario cause it gives people the option to choose you know that*’*s important*”. (*MM04*, *Unknown Drinking Status*)

One young Māori male made reference to the success of a recent depression campaign using a high profile sports personality:

“..*really like that*, *those ads you know just cause they*’*re real I suppose*, *he*’*s a real*, *an aspect of reality you know real*, *they*’*re not a reality TV show thing you know but actually talking from the heart I guess*”. (*MM04*, *Unknown Drinking Status*)

Another participant described the need for the messages to come from an identifiable person:

“*If you don*’*t know who it*’*s coming from*, *it*’*s hard to respect a text coming from an anonymous source*…” (*NE03*, *Hazardous Drinker*)

The idea that the ‘face of the initiative’ should be a celebrity who has overcome alcohol problems was raised:

“*I think ideally the message should be from somebody who is well known and I suppose my personal preference would be from somebody who I knew had an alcohol problem and overcome it so they*’*re sort of saying I know what it*’*s like I*’*ve been there but you know hang in there you can overcome this*”. (*NE16*, *Non*-*Hazardous Drinker*)

Others indicated they didn’t think celebrity endorsement was genuine or believable, with one young male noting:

“*Yeah*, *that*’*s never done anything for me*…*like it*’*s not like they*’*re endorsing a product or something*, *but when they are sort of in the spotlight for something*, *it just seems like they*’*re doing it for the publicity rather than because they actually care. Even if they do care*, *it just seems like they don*’*t*”. (*NE20*, *Non*-*Hazardous Drinker*)

Another young male felt messages would be most effective if sent from mates, making the following observation:

“…*obviously the best people to get a text like that from would be your friends. They*’*ve probably got like they*’*ve got the most power in terms of telling you to stop drinking like you know we tell people who get a bit too sloppy when they*’*re drunk like in the morning say* ‘*oh get a bit sloppy last night*’…” (*NE03*, *Hazardous Drinker*)

One middle-aged participant recommended providing the recipients choice in who (from a panel of potential sources) they would with to receive messages from.

### Text messaging features

There was more support for text than video or picture messages (Table 1). One male participant commented:

“*I guess not everyone has a pxt phone. I think most people have*, *but I guess a lot of people still don*’*t have a pxt phone*, *so they wouldn*’*t get the message*”. (*NE20*, *Non*-*Hazardous Drinker*)

### Timing of messages

Strong views were expressed regarding the timing of when the proposed intervention is delivered. Most people recommended that the number of messages should be restricted to once or twice a week focusing on the early evening hours on peak drinking days such as Thursday to Sunday. A middle-aged male pointed out the importance of delivering messages before drinking commences:

“*And often I don*’*t know they might be too inebriated really by then cause I mean I used to drink*, *you know when I drink heavily as a youngster but it*’*s very very quick*, *I*’*d have one glass of the other feeling better and thinking that the more I drank the better I*’*d feel and then I*’*d be absolutely wasted so there*’*s a certain threshold where any message isn*’*t going to be of any use you know*, *you*’*ve gone beyond return*”. (*NE17*, *Non*-*Hazardous Drinker*)

In contrast, one female participant thought after the weekend was a better time:

“*You could also do something like after the weekend something like you know if you know how was your drinking this weekend if you have any regrets or something give us a call and we can talk about it* …” (*NE15*, *Non*-*Hazardous Drinker*)

Another signalled the messages needed to come when you had the time to read them:

“…*it would definitely have to be a specific time*, *you know not in a busy time of the work day so they would just be I*’*m too busy to even look at this*, *I think timing is really important*”. (*MM04*, *Unknown Drinking Status*)

#### Theme four: intervention content issues

Issues that were raised by participants during the course of interviews regarding the content of the proposed intervention clustered into four lower level themes: tone of the messages, message ideas, cultural relevance, and concerns regarding the invasion of privacy and confidentiality.

### Message tone

Participants believed the tone of the messages needed to be ‘positive and supportive’ or ‘bright and breezy’ and not judgmental in order to make a difference to drinkers’ behavior as illustrated by these comments:

“*I think it would be akin to having a nagging mother*, *you start to ignore what they say and then start to want to do the opposite*”. (*NE02*, *Non*-*hazardous Drinker*)

“*I guess being too judgmental*, *yeah*, *and too sort of confrontational. I mean*, *you need a bit of it*, *but I guess you*’*d turn people away if you just jumped on them*”. (*NE20*, *Non*-*Hazardous Drinker*)

“*I think it*’*d be good*, ‘*cause it*’*d make you feel a bit guilty about your drinking and it would make you aware of it. And the idea that someone else is aware of it too*, *you know*, *like the idea of someone saying to you*, “*What you*’*re about to do is a problem*,” *or something like that*, *it*’*s kind of confrontational without being*, *you know*, *rude*, *but it is a little bit in your face*”. (*NE20*, *Non*-*Hazardous Drinker*)

There was a suggestions that the help and support provided could be similar to that offered by telephone support services for people wanting to quit smoking. A middle-aged participant suggested the following:

“… *they probably may have had incidences before and are wanting a helping hand*, *almost like Quitline. Going out tonight don*’*t you know*, *pace you know that sort of thing* …*so it*’*s basically people knowing that they*’*ve got a problem and they just*, *if their friends don*’*t remind them their cell phone will*”. (*NE17*, *Non*-*Hazardous Drinker*)

A common theme was the importance of keeping the messages simple and focusing on the key issues as these comments from young Māori males suggest:

“*Yeah*, *like keep it simple so people can understand and relate to whatever information you*’*re giving them*, *you know*…” (*MM06*, *Hazardous Drinker*)

“*Just think what you*’*re going to do before you do it*, *yeah don*’*t drink and drive that*’*s a big one for me*”. (*MM03*, *Hazardous Drinker*)

The potential for misinterpretation of written messages sent via phone was of concern to some participants:

“*Language is an important thing sort of*, *it*’*s quite a tricky one. How you word it would be important*”. (*MM04*, *Unknown Drinking Status*)

“*The problem with* …*trying to converse*, *communicate over a cell phone text is really difficult cause it could be read in so many different ways because people interpret the language that is used in so many different ways*”. (*MM04*, *Unknown Drinking Status*)

### Message ideas

A number of participants suggested the proposed intervention should include practical information such as: how to seek help for alcohol issues, to advise drinkers to give their car keys to a sober friend, or to promote non-alcohol related activities as a substitute for alcohol-related activities. A female participant in her thirties explained:

“*I mean I personally wouldn*’*t want just you know a text saying you know don*’*t drink*, *you know I*’*d want it to be something tangible that I could*, *you know*, *that was practical that I could use*…” (*NE15*, *Non*-*Hazardous Drinker*)

This young male made the following suggestion regarding the type of advice that could be given:

“…*if I got say a text before I went out saying do you need to drink 20 standard drinks tonight*, *do you need to spend* $*100 going out on alcohol tonight*? *You know will your drinking make you*, *will it lead to a bad decision*…” (*NE03*, *Hazardous Drinker*)

The idea that messages should contain relevant facts and statistics was a common theme among younger people:

“…*you could give some statistical information even*, *you know*, “*Drink driving causes so much damage per year*,” *or costs so much to the country or*, “*This many drink drivers have been caught on the road*, *don*’*t make it you*” …” (*NE06*, *Non*-*hazardous Drinker*)

“*I reckon if you could have that in terms of excessive drinking and what happens*, *you know*? *Like if you got a pxt of* – *I don*’*t know a liver* … *something like that. Yeah*, *that could work*”. (*NE20*, *Non*-*Hazardous Drinker*)

### Cultural relevance

Participants emphasised the importance of the messages being culturally relevant and relating to issues that are important and relevant for Māori. The following two comments from a middle-aged male illustrate this point:

“*Well to me when I think life is precious you know that sort of thing*, *I guess being Maori as well the first thing that comes to mind is family you know*, *people around me*, *my spirituality and the land in which I live you know*, *those are the things that are precious to me*”. (*MM04*, *Unknown Drinking Status*)v

“*Like those things of you know family*, *you know their spiritual life I mean the people around me*, *the land around me*… *for me anyway like makes*, *yeah me want to change and take responsibility for my life*, *it*’*s about taking responsibility you know owning it*, *taking responsibility for your actions*” (*MM04*, *Unknown Drinking Status*)

### Invasion of privacy and confidentiality

Concerns were raised that texts could be seen as in invasion of privacy or intrusive and eventually ignored:

“*I suppose overstepping the mark*, *you know*, *overstepping those boundaries of privacy and it*’*s my text and did I invite*… *you know*”. (*NE10*, *Non*-*hazardous Drinker*)

“*I don*’*t know whether it would just fall in to the junk mail category*, *something that people immediately ignore and then get annoyed about because it*’*s intrusive*”. (*NE02*, *Non*-*hazardous Drinker*)

The issue of confidentiality was raised by a number of younger participants:

“*People might be embarrassed if I*’*ve got my phone out if something comes up like how much did you drink last night I*’*d be like oh embarrassed*”. (*NE15*, *Non*-*Hazardous Drinker*)

“…*cause your phone is quite personal*, *so you don*’*t want it to be messages that you don*’*t want anyone to see*”. (*NE18*, *Non*-*hazardous Drinker*)

One female participant said they would not take part in a study if their name would be used in texts, stating:

“*Probably if you knew your name was gonna be attached to it and it was not gonna be*, *you know*, *no confidentiality*”. (*NE07*, *Non*-*hazardous Drinker*)

## Discussion

This study investigated the appropriateness, appeal, acceptability, and potential content of a BI delivered via text messaging to reduce harmful drinking behaviours among admitted trauma patients. The findings from this small sample of adult trauma in-patients (one quarter of whom were hazardous drinkers) indicate that a tailored mobile phone text messaging intervention for hazardous drinking could be effective, and that the majority of patients interviewed would enrol in such a service. Participants indicated that text messages providing information about alcohol could be effective in reducing hazardous drinking and related harms. Factors that participants felt would make them more receptive to an m-health intervention included: an evidence-based intervention, a readiness to change on the part of participants, and ease of use. The most common barriers to participation identified were: the timeliness of messages (too frequent, annoying), and that the messages could be easily ignored. Participants also raised the potential concerns regarding perceived breaches of privacy and confidentiality.

Opinions were mixed regarding the potential value of using a celebrity as the ‘face’ of the intervention, and the provision of incentives for participation. Text messages were identified as the preferred format for delivery of a tailored m-health intervention. In relation to content, participants felt the tone of the message needed to be non-judgemental, and the messages needed to be both informative with respect to practical advice in relation to reducing drinking and factual with respect to the consequences of drinking.

This research has a number of strengths including the alignment of this research with Whittaker et al.’s first (Formative Research) and second (Pretesting) steps in the research and evaluation process for developing m-health interventions [27]. The current study aligns with use of the general inductive approach that uses detailed readings of raw data to develop concepts, themes, or a model through suppositions made from the data by the researcher [30]. The availability of Māori and Pacific interviewers may have increased the likelihood of these often hard to reach populations agreeing to take part in the research. Māori attitudes to m-health messaging have been largely unexplored and this research now extends the available literature.

The findings need to be considered in light of several limitations. The low numbers of hazardous drinkers in this study (7/30) is a major limitation of the study. The purposeful sampling approach used means the findings have generated participants’ ‘insights and in-depth understanding’ in relation to the proposed intervention rather than ‘empirical generalisations’ about all hazardous drinkers. However, it was reassuring to note that more than half of these participants indicated they would sign up for a free m-health service that delivered alcohol related messages, and of those half identified as being of Māori descent. The numbers of Māori and Pacific participants were lower than anticipated due to numbers admitted during the recruitment period. The absence of Asian specific interviewers may have impacted on the likelihood of this population group to participate in the research. Participants noted the importance of ensuring the cultural relevance of messages, an aspect particularly noted by Māori respondents. While the findings overall cannot be generalised to all communities involved, the heterogeneous ethnic composition of participants provides a range of perspectives relevant to the study objectives.

Social desirability bias may have been an issue in the present study as participants may have been inclined to respond favourably to the proposed intervention [32-34]. We attempted to minimise this bias by asking questions in an open-ended way, particularly prompting the likelihood that the intended approach may not be acceptable. The interviewers were largely conducted by research assistants who were not the primary investigators and were not directly involved with the intervention/project development plan to encourage eliciting a broader range of views.

In addition, we have no way of confirming if participants who indicated they would be agreeable to taking part in a text messaging based intervention would actually take part if invited to enrol in a randomised controlled trial.

Participants in the present study identified too frequent messaging as a potential barrier to participation. This finding is consistent with concerns raised in a study assessing the effectiveness using handheld computers to deliver personalised feedback on college students’ alcohol consumption that found one of the most common complaints from participants was they felt they received too many messages (at least once every two days), and that there was too much repetition [35]. In contrast, a study by Moore et al. exploring the feasibility of delivering text message alcohol related intervention to University students found the frequency of messages was not perceived as a barrier [36]. A feasibility study conducted by Crombie et al. looked at the appeal of a text based intervention to address alcohol related harm in disadvantaged men, found four texts per week maximum was appropriate [37].

Participants in this study, suggested messages should be balanced with respect to containing practical advice in relation to reducing drinking and factual with respect to the consequences of drinking. This sentiment is echoed in the study by Moore where participants suggested balancing negative and positive messaging [36].

Concerns around privacy and confidentiality of text messages were raised by participants in the present study. In contrast, the study by Moore et al. found their participants perceived alcohol related messages delivered by text to be ‘secure and private’ [36].

A recent review which affirmed the positive benefits of behaviour change interventions delivered by text messages identified features associate with success as: messaging interactivity and tailoring content [38]. Messaging interactivity includes the ability to send updates of participant’s progress if requested, and answer participant derived questions, and SOS message options. Ways of tailoring messages included using the person’s name or nickname, making reference to behavioural preferences, participants’ goals, and barriers and enablers to achieving their goals.

Renner et al. conducted a randomised controlled trial among healthy volunteers to establish the effectiveness of a BI which included a schedule of alcohol harm reduction text messages that participants had developed for themselves [39]. The study found that participants created messages with a diversity of content that bore little resemblance to commonly used BI messages.

## Conclusions

The results of this study provide evidence that suggests adult trauma patients would be receptive to an m-health intervention to address hazardous drinking. A large scale randomised controlled trial to test the effectiveness of an m-health intervention for alcohol misuse is warranted. In the next phase of this research, the team will draw on expertise from social marketing, community drug and alcohol services, m-health, trauma care clinicians, cultural advisors, and lay representatives to refine the methodology for a trial including testing the text-messages. The format, frequency, and timing of the text messages will in part be informed by the findings of this study.

## Abbreviations

BI: Brief intervention; BIs: Brief interventions; ED: Emergency department; m-health: The delivery of health information via mobile phones; SMS: Short message service.

## Competing interests

The authors report no conflicts of interests.

## Authors' contributions

BK developed the study methodology, led the analysis, and drafted the manuscript. ES and KR collected the data, assisted with the analysis and contributed to the preparation of the draft. SA developed the study methodology, oversaw the project, and contributed to the preparation of the manuscript. All authors read and approved the final manuscript.

## Supplementary Material

Additional file 1 **Feasibility of a mobile phone based alcohol intervention.** Individual Interview - question guide. Click here for file
