# Supplementary material for: Perceptions of adult trauma patients on the acceptability of text messaging as an aid to reduce harmful drinking behaviours
Source: BMC Res Notes. 2014 Jan 4;7:4. doi: 10.1186/1756-0500-7-4 (PMC3884009; doi:10.1186/1756-0500-7-4)
Supplement: Additional file 1 — Feasibility of a mobile phone based alcohol intervention. Individual Interview - question guide. [file 1756-0500-7-4-S1.pdf]

Feasibility of a mobile phone based alcohol intervention

Individual Interview - question guide

Hello I am .... a researcher from the University of Auckland. We would like to get some feedback on a research study we are hoping to run will test the effectiveness of an innovative multi-media mobile phone intervention targeting people admitted to hospital as a result of an injury. The goal of the intervention is to alter the way people drink alcohol to reduce the harms associated with alcohol use. The intervention aims to: overcome barriers that hamper interventions targeting hard to reach groups (particularly youth), address health inequities amount Maori, pacific and low socio-economic populations.

We are interested in getting feedback from a small group of patients to see if they think an intervention such as the one we are proposing would be effective, and to see what things would make people more or less likely to take part.

Show patients the Information Sheet, allow them to time to read it/or read it to them if they prefer, answer any questions. Ask if they would like to take part if they agree, obtain written consent. If they decline to take part, thank them for their time.

Thank you for agreeing to take part in this study. I will begin by asking some open ended questions, and then there is a brief written questionnaire for you to complete.

Are you comfortable? Do you need a drink? Commence interview START TAPE

**Individual Interview guide**	
Question One<br>*Guide – Perceptions of interventions to reduce hazardous drinking to prevent further injury*	What previous media campaigns (TV, radio, newspaper etc) if any do you think have made an impact on the way you or your friends drink?<br><br>If they have had an impact what about them has done this?
Question Two<br>*Guide – Perceptions of interventions to reduce hazardous drinking to prevent further injury*	From your view point, what kind of new measures (interventions) do you think could be effective in reducing hazardous drinking and further alcohol related harm?<br><br>How effective do you think mobile phone messages would be in delivering information regarding alcohol? *(How would you feel about getting a text message)?*<br><br>Would you sign up if there was a free service?
Question Three<br>*Guide – Perceived acceptability of m-health intervention; e.g. how it could be helpful, potential downsides or pitfalls with this method of delivery, what sorts of messages would be useful, would social support be a useful component, and possible methods of interactivity*	Would you personally see them as being helpful (*what would they say*) and why?<br><br>What do you see as being the potential pitfalls?
Question Four<br>*Guide – Perceived enablers to an m-health intervention.*	What do you think researchers need to bear in mind to make someone like you more likely to take part in a study like the one we are proposing?
Question Five<br>*Guide – Perceived barriers to an m-health intervention.*	What things do you think might make someone like you less likely to take part in a study like the one we are proposing?
I would now like to invite you to complete this brief written questionnaire which covers areas like the type of cell-phone you have, how often you use it, what network you are on etc. And some questions about your alcohol use	

Thank you very much for taking part. (Stop tape, enter participant details in Study Log)
